# miR-107 inhibition upregulates CAB39 and activates AMPK-Nrf2 signaling to protect osteoblasts from dexamethasone-induced oxidative injury and cytotoxicity

**DOI:** 10.18632/aging.103341

**Published:** 2020-06-11

**Authors:** Yu Zhuang, Shouguo Wang, Haodong Fei, Feng Ji, Peng Sun

**Affiliations:** 1 Department of Orthopedics, The Affiliated Huaian No.1 People’s Hospital of Nanjing Medical University, Huaian, China

**Keywords:** miR-107, CAB39, osteoblasts, dexamethasone, AMPK-Nrf2 signaling

## Abstract

To human osteoblasts dexamethasone (DEX) treatment induces significant oxidative injury and cytotoxicity. Inhibition of CAB39 (calcium binding protein 39)-targeting microRNA can induce CAB39 upregulation, activating AMP-activated protein kinase (AMPK) signaling and offering osteoblast cytoprotection. Here we identified a novel CAB39-targeting miRNA: the microRNA-107 (miR-107). RNA-Pull down assay results demonstrated that the biotinylated-miR-107 directly binds to *CAB39 mRNA* in OB-6 human osteoblastic cells. Forced overexpression of miR-107, by infection of pre-miR-107 lentivirus or transfection of wild-type miR-107 mimic, largely inhibited CAB39 expression in OB-6 cells and primary human osteoblasts. Contrarily, miR-107 inhibition, by antagomiR-107, increased its expression, resulting in AMPK cascade activation. AntagomiR-107 largely attenuated DEX-induced cell death and apoptosis in OB-6 cells and human osteoblasts. Importantly, osteoblast cytoprotection by antagomiR-107 was abolished with AMPK in-activation by AMPKα1 dominant negative mutation, silencing or knockout. Further studies demonstrated that antagomiR-107 activated AMPK downstream Nrf2 cascade to inhibit DEX-induced oxidative injury. Conversely, Nrf2 knockout almost abolished antagomiR-107-induced osteoblast cytoprotection against DEX. Collectively, miR-107 inhibition induced CAB39 upregulation and activated AMPK-Nrf2 signaling to protect osteoblasts from DEX-induced oxidative injury and cytotoxicity.

## INTRODUCTION

Sustained and/or excessive long-term usage of Dexamethasone (DEX) could induce osteoporosis or even osteonecrosis [1]. To cultured human osteoblasts or osteoblastic cells DEX treatment will induce profound cytotoxicity and cell apoptosis [2–5]. Furthermore, in the bones of DEX-taking patients significant osteoblast cell apoptosis and decreased number of viable osteoblasts were detected [6, 7]. Our group has been dedicated to understanding the signaling mechanisms of DEX-induced osteoblast cytotoxicity, and to developing novel and efficient strategies to overcome them [2–5].

AMP-activated protein kinase (AMPK) is a key energy senor, regulating energy metabolic balance and homeostasis at both cellular and physiological levels [8]. Activated AMPK can promote cell survival under stress conditions [9]. By directly phosphorylating autophagy-related proteins (including ULK1, Beclin-1, and Vps34), AMPK initiates cytoprotective autophagy [10, 11]. Under oxidative stress AMPK activation can suppress reactive oxygen species (ROS) production and oxidative injury [5, 12–15]. AMPK is vital for maintain nicotinamide adenine dinucleotide phosphate (NADPH) homeostasis [5, 12–15]. Additionally, AMPK will activate its potential downstream Nrf2 signaling to alleviate oxidative injury [16, 17]. AMPK activation also blocks mammalian target of rapamycin complex 1 (mTORC1) signaling, favoring cell survival under energy crisis conditions [18, 19]. Thus, AMPK is a pro-survival signaling in stressed human cells.

Our previous studies have suggested that forced activation of AMPK, genetically or pharmacologically, can offer significant osteoblast cytoprotection. For example, compound 13, an alpha1 selective AMPK activator, inhibited DEX-induced osteoblast cell death and apoptosis [5]. The benzimidazole derivative compound 991, a novel and highly-efficient AMPK activator, attenuated DEX-induced oxidative injury and osteoblast cytotoxicity [20]. MicroRNA-429, which activated AMPK signaling through silencing AMPKα1 phosphatase protein phosphatase 2A, protected human osteoblasts from DEX [21]. Furthermore, GSK621, a novel AMPK activator, activated AMPK signaling and protected osteoblasts from hydrogen peroxide (H_2_O_2_) [13].

CAB39 (calcium binding protein 39) is a component of the trimeric LKB1-STRAD-CAB39 complex, required for the stabilization of STRAD to LKB1 binding. It promotes LKB1 translocation from the nuclei to the cytoplasm [22]. LKB1-STRAD-CAB39 complex activates AMPK signaling by phosphorylating AMPKα1 at Thr-172 [22]. MicroRNAs (miRNAs) are a large family of small (~22 nucleotides) non-coding RNAs (ncRNAs), regulating gene expression at the translational and post-transcriptional levels [23, 24]. miRNAs bind to 3′-untranslated regions (3′-UTRs) of specific mRNAs, thus inhibiting their translation and/or inducing them degradation [23, 24]. Previous studies have shown that inhibition of CAB39-targeting microRNA (*i.e.* miR-451) induced CAB39 upregulation, thus activating AMPK signaling [22, 25, 26]. The results of the present study identified a novel CAB39-targeting miRNA, microRNA-107 (miR-107). miR-107 inhibition upregulated CAB39 and activated AMPK signaling, protecting osteoblasts from DEX-induced oxidative injury and cytotoxicity.

## RESULTS

### miR-107 targets and silences CAB39 in osteoblasts

**Figure 1:**
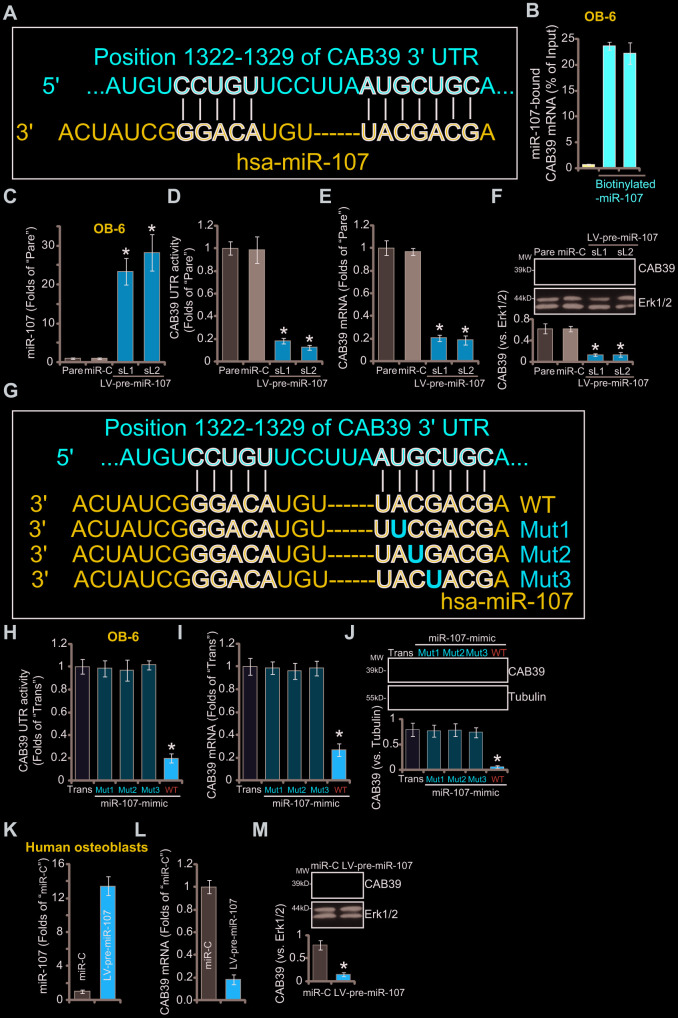
**miR-107 targets and silences CAB39 in osteoblasts.** The bioinformatics analyses show that miR-107 putatively targets 3’-UTR of *human CAB39* (at position of 1322-1329) (**A**). The RNA-Pull down assay confirmed the binding between the biotinylated-miR-107 and *CAB39 mRNA* (normalized to the input control) (**B**). Stable OB-6 cells with pre-miRNA-107 lentivirus (LV-pre-miR-107-sL1/sL2, two stable cell lines) or non-sense microRNA control lentivirus (“miR-C”, same for all Figures), as well as the parental control OB-6 cells (“Pare”, same for all Figures), were cultured, expression of miRNA-107 and CAB39 was tested by qPCR (**C** and **E**) and Western blotting (**F**) assays, with relative CAB39 3’-UTR luciferase activity (**D**) examined as well. OB-6 cells were transfected with 500 nM of the applied miR-107 mimics (sequences listed in **G**) for 48h, CAB39 3’-UTR luciferase activity (**H**) and its expression (**I** and **J**) were tested. The primary human osteoblasts were infected with pre-miRNA-107 lentivirus (LV-pre-miR-107) or miR-C, after 48h expression of listed genes was shown (**K**–**M**). Data were mean ± standard deviation (SD, n=5). “Trans” stands for the transfection reagent control (**H**–**J**). * p<0.05 *vs.* “miR-C”/“Trans” cells. Each experiment was repeated three times and similar results were obtained.

First we explored miRNAs that can possibly target CAB39. TargetScan (V7.2, http://targetscan.org, V7.2) [27] was first consulted. Multiple miRNAs specifically targeting the 3’-UTR of human CAB39 were indentified, that were further verified by other miRNA databases, including miRbase and miRDB. The bioinformatics analyses have identified that miR-107 putatively targets 3’-UTR of CAB39 (at position of 1322-1329) (Figure 1A). The context^++^ score for miR-107-CAB39 3’-UTR binding is -0.53, with the score percentage of 99% (from TargetScan). These parameters indicated a high percentage of binding between the two [27]. By performing the RNA-Pull down assay in OB-6 human osteoblastic cells, we show that the biotinylated-miR-107 directly associated with *CAB39 mRNA* (Figure 1B). The streptavidin-coated magnetic beads (“Beads”), as expected, did not bind to *CAB39 mRNA* (Figure 1B).

To verify that miR-107 is a CAB39-targeting miRNA, the lentivirus expressing pre-miRNA-107 (LV-pre-miR-107) was constructed. The virus was transduced to OB-6 osteoblastic cells. Subjected to puromycin selection two stable cell lines, LV-pre-miR-107-sL1/sL2, were established, showing over 20-folds increase of mature miR-107 expression (*vs.* control cells, Figure 1C). Importantly, forced overexpression of miR-107 significantly inhibited CAB39 3’-UTR luciferase activity in OB-6 cells (Figure 1D). Furthermore, expression of *CAB39 mRNA* (Figure 1E) and protein (Figure 1F) was potently decreased in LV-pre-miR-107-expressing OB-6 cells. These results implied that ectopic miR-107 overexpression silenced CAB39 in OB-6 cells. The non-sense microRNA control lentivirus, or miR-C, did not affect miR-107 and CAB39 expression in OB-6 cells (Figure 1C–1F).

To further support our hypothesis, we synthesized three mutant miR-107 mimics, containing mutations at the binding sites to the CAB39 3'-UTR (see sequences in Figure 1G). As shown, in OB-6 cells transfection of the three mutants, “Mut1/2/3”, failed to affect CAB39 3’-UTR luciferase activity (Figure 1H) and its expression (mRNA/protein, Figure 1I and 1J). Contrarily, transfection of same concentration of the wild-type (“WT”) miR-107 mimic resulted in robust inhibition of CAB39 3’-UTR luciferase activity (Figure 1H) and its expression (Figure 1I and 1J). These results further confirm that miR-107 targets and silences CAB39 in OB-6 cells. In the primary human osteoblasts, infection of LV-pre-miR-107 resulted in miR-107 overexpression (Figure 1K), but *CAB39 mRNA* (Figure 1L) and protein (Figure 1M) downregulation.

### miR-107 inhibition causes CAB39 upregulation and AMPK signaling activation in osteoblasts

**Figure 2:**
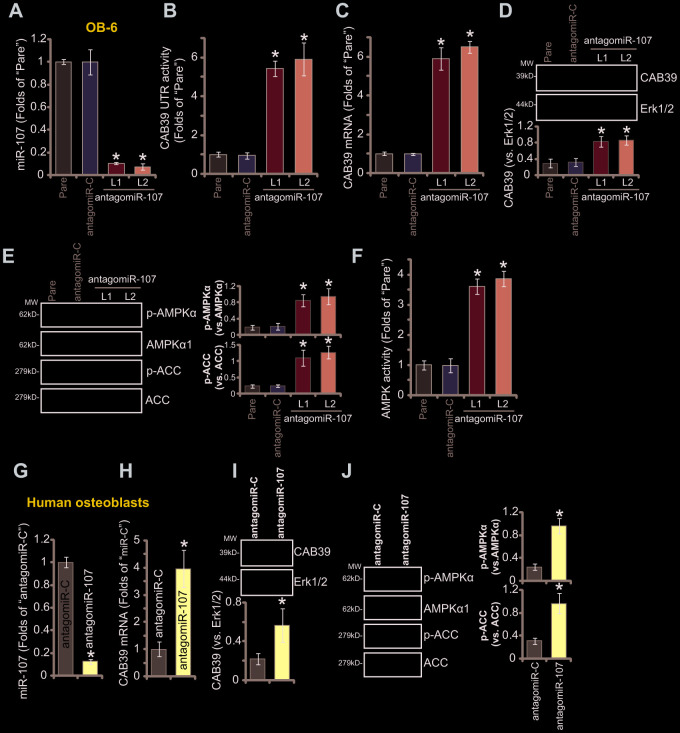
**miR-107 inhibition causes CAB39 upregulation and AMPK signaling activation in osteoblasts.** Stable OB-6 cells with pre-miRNA-107 anti-sense lentivirus (antagomiR-107-L1/L2, two stable cell lines) or control anti-sense lentivirus (antagomiR-C), as well as the parental OB-6 cells were cultured, expression of mature miRNA-107, CAB39 and AMPK signaling proteins was tested by qPCR (**A** and **C**) and Western blotting (**D** and **E**) assays, with relative CAB39 3’-UTR luciferase activity (**B**) and AMPK activity (**F**) examined as well. The primary human osteoblasts were infected with antagomiR-107 lentivirus or antagomiR-C lentivirus for 48h, expression of listed genes was shown (**G**–**J**). Data were mean ± standard deviation (SD, n=5). * p<0.05 *vs.* “antagomiR-C” cells. Each experiment was repeated three times and similar results were obtained.

To suppress miR-107, the pre-miR-107 anti-sense lentivirus, antagomiR-107, was transduced to cultured OB-6 cells. Two stable cell lines, antagomiR-107-L1/L2, were established with selection by puromycin-containing medium. The mature miR-107 levels decreased over 90% in the stable cells (Figure 2A), where the CAB39 3’-UTR luciferase activity (Figure 2B) and its expression (Figure 2C and 2D) were elevated. Therefore miR-107 inhibition upregulated CAB39 in OB-6 cells.

CAB39 is a scaffold protein of LKB1, the latter is the upstream kinase of AMPK [22, 28, 29]. Increased CAB39 expression could possibly provoke AMPK signaling activation [22, 25, 28, 29]. In the present study we show that miR-107 inhibition activated AMPK signaling in OB-6 cells, as phosphorylation (“p-”) of AMPKα1 (Thr-172) and its major downstream target protein acetyl-CoA carboxylase (ACC, Ser-79) was significantly enhanced in antagomiR-107-expressing OB-6 cells (Figure 2E). Furthermore, the AMPK activity was augmented with miR-107 inhibition (Figure 2F). In the primary human osteoblasts antagomiR-107 similarly resulted in reduction of mature miR-107 (Figure 2G) but upregulation of CAB39 (Figure 2H and 2I). Additionally significant AMPKα1-ACC phosphorylation was detected in antagomiR-107-expressed osteoblasts (Figure 2J), indicating AMPK signaling activation. The anti-sense control lentiviral construct, antagomiR-C, did not affect CAB39 expression and AMPK signaling (Figure 2A–2J).

### miR-107 inhibition protects osteoblasts from DEX-induced cell death and apoptosis

**Figure 3:**
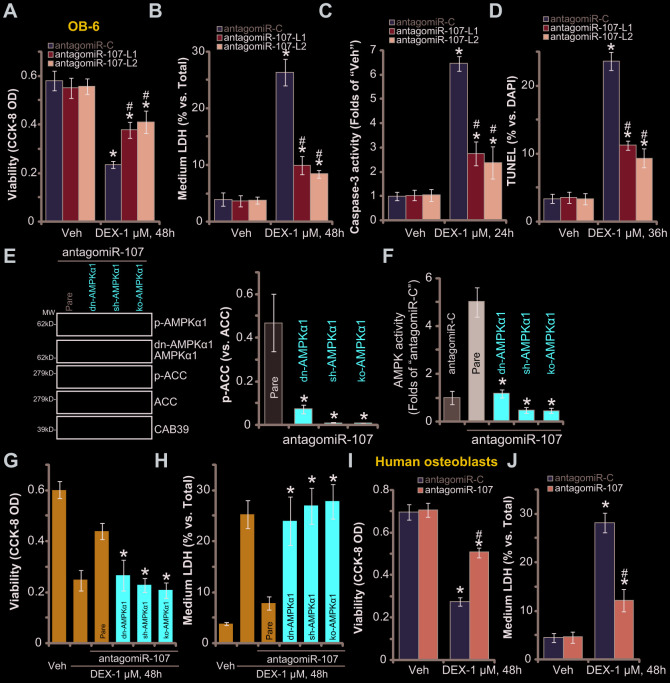
**miR-107 inhibition protects osteoblasts from DEX-induced cell death and apoptosis.** OB-6 cells (**A**–**D**) or primary human osteoblasts (**I**–**J**) with pre-miRNA-107 anti-sense lentivirus (antagomiR-107) or control anti-sense lentivirus (antagomiR-C), were treated with DEX (1 μM) or the vehicle control (“Veh”) for indicated time periods, cell viability (CCK-8 OD, A and I), cell death (medium LDH release, **B** and **J**), caspase-3 activity (**C**) and cell apoptosis (nuclear TUNEL staining, **D**) were tested. The stable OB-6 osteoblastic cells, with the dominant negative AMPKα1 (dn-AMPKα1, T172A) construct, the lentiviral AMPKα1 shRNA (sh-AMPKα1), the CRISPR-Cas-9-AMPKα1 KO plasmid (ko-AMPKα1), as well as the parental control cells (“Pare”) were infected with antagomiR-107 lentivirus for 48h, expression of listed proteins (**E**) and the relative AMPK activity (**F**) were tested; Alternatively, cells were treated with DEX (1 μM) or the vehicle control (“Veh”) for another 48h, cell viability (CCK-8 OD, **G**) and cell death (medium LDH release, **H**) were tested. Data were mean ± standard deviation (SD, n=5). * p<0.05 *vs.* “Veh” treatment in “antagomiR-C” cells (**A**–**D**, **I**–**J**). ^#^ p<0.05. *vs.* “DEX” treatment in “antagomiR-C” cells (**A**–**D**, **I**–**J**). * p<0.05 *vs.* “Pare” cells (**E**–**H**). Each experiment was repeated three times and similar results were obtained.

Studies have demonstrated that forced activation of AMPK signaling can efficiently protect osteoblasts from DEX-induced cell death and apoptosis [5, 20, 21, 30, 31]. Since antagomiR-107 upregulated CAB39 and activated AMPK signaling, we investigated its activity in DEX-treated osteoblasts. As shown, in control OB-6 cells with antagomiR-C DEX treatment induced potent cell viability (CCK-8 OD) reduction (Figure 3A), cell death (medium LDH release, Figure 3B), caspase-3 activation (Figure 3C) and cell apoptosis (nuclear TUNEL staining increase, Figure 3D). Importantly, in OB-6 cells with antagomiR-107, DEX-induced cytotoxicity (Figure 3A and 3B) and apoptosis (Figure 3C and 3D) were significantly alleviated. These results implied that miR-107 inhibition protected OB-6 cells from DEX-induced cell death and apoptosis.

To study the link between antagomiR-107-induced AMPK signaling activation and anti-DEX osteoblast cytoprotection, genetic strategies were utilized to block AMPK activation. First, the dominant negative AMPKα1 (“dn-AMPKα1”, T172A) ([5, 21]) was transfected to OB-6 cells, with selection stable cells established. Western blotting assay results, Figure 3E, confirmed the mutant AMPKα1 expression (Flag-tagged) in stable cells. Second, the AMPKα1 shRNA lentiviral particles were added to OB-6 osteoblastic cells, resulting in significant AMPKα1 downregulation (“sh-AMPKα1”, Figure 3E). Furthermore the CRISPR-Cas-9 strategy [32] was applied to knockout AMPKα1 in OB-6 cells (“ko-AMPKα1”, Figure 3E). As demonstrated, antagomiR-107-induced AMPK activation was largely inhibited in OB-6 cells with dn-AMPKα1, sh-AMPKα1 or ko-AMPKα1. AMPK cascade activation was tested by AMPKα1/ACC phosphorylation (Figure 3E) and AMPK activity increase (Figure 3F). Significantly, antagomiR-107-induced OB-6 cytoprotection against DEX was almost reversed by AMPKα1 mutation, silencing or KO (Figure 3G and 3H). AntagomiR-107 was largely ineffective against DEX-induced viability reduction (Figure 3G) and cell death (Figure 3H) when AMPKα1 was mutated, silenced or depleted (Figure 3G and 3H). These results clearly show that AMPK activation is required for antagomiR-107-induced OB-6 cytoprotection against DEX. In the primary human osteoblasts, antagomiR-107 infection largely inhibited DEX-induced viability reduction (Figure 3I) and cell death (Figure 3J). Together, miR-107 inhibition protected osteoblasts from DEX-induced cell death and apoptosis.

### miR-107 inhibition activates Nrf2 signaling and alleviates DEX-induced oxidative injury in osteoblasts

**Figure 4:**
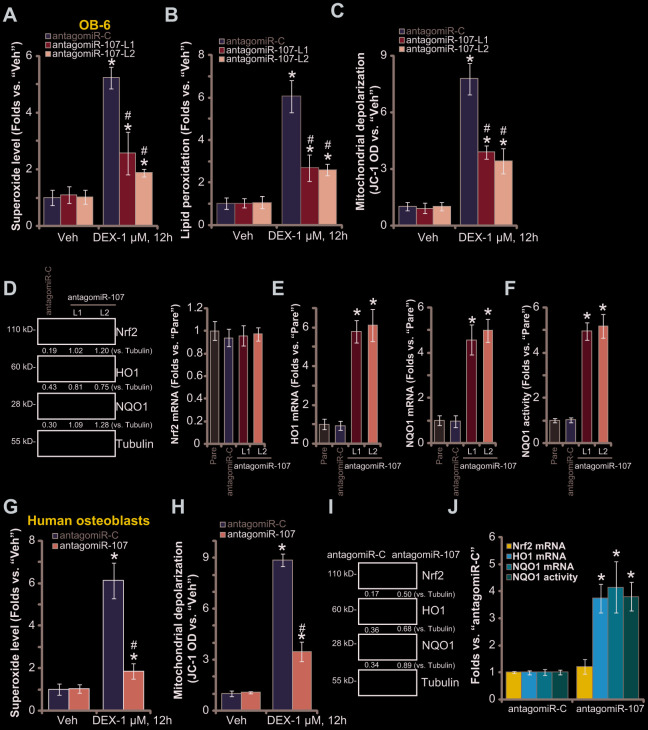
**miR-107 inhibition alleviates DEX-induced oxidative injury in osteoblasts. OB-6 cells** (**A**–**C**) or primary human osteoblasts (**G** and **H**) with pre-miRNA-107 anti-sense lentivirus (antagomiR-107) or control anti-sense lentivirus (antagomiR-C), were treated with DEX (1 μM) or the vehicle control (“Veh”) for 12h, cellular superoxide contents (**A** and **G**), lipid peroxidation levels (**B**) and mitochondrial depolarization (JC-1 green fluorescence intensity, **C** and **H**) were tested. Expression of listed Nrf2 pathway genes in OB-6 cells and primary human osteoblasts, with antagomiR-107 or antagomiR-C, was shown (**D**, **E**, **I** and **J**), with NQO1 activity tested as well (**F** and **J**). Data were mean ± standard deviation (SD, n=5). * p<0.05 *vs.* “Veh” treatment in “antagomiR-C” cells (**A**–**C**, **G** and **H**). ^#^ p<0.05. *vs.* “DEX” treatment in “antagomiR-C” cells (**A**–**C**, **G** and **H**). * p<0.05 *vs.* “Pare” cells (**E** and **F**). Each experiment was repeated three times and similar results were obtained.

DEX treatment induces profound ROS production and oxidative injury, responsible for osteoblast cell death and apoptosis [20, 30, 33–35]. Since antagomiR-107 activated AMPK signaling and protected osteoblasts from DEX-induced cell death and apoptosis, we next studied its activity on DEX-induced oxidative injury. As shown, in the antagomiR-C-expressing OB-6 cells DEX treatment induced robust oxidative injury, evidenced by superoxide accumulation (Figure 4A), increased lipid peroxidation (Figure 4B) and mitochondrial depolarization (JC-1 green fluorescence accumulation, Figure 4C). Significantly, miR-107 inhibition by antagomiR-107 largely attenuated DEX-induced oxidative injury in OB-6 cells (Figure 4A–4C). Further studies demonstrated that miR-107 inhibition induced Nrf2 signaling cascade activation, resulting in Nrf2 protein stabilization (Figure 4D), as well as increased *HO1*-*NQO1* mRNA-protein expression (Figure 4E) and NOQ1 activity increase (Figure 4F). *Nrf2 mRNA* levels were however unchanged (Figure 4D).

In the primary human osteoblasts, antagomiR-107 infection also alleviated DEX-induced superoxide accumulation (Figure 4G) and mitochondrial depolarization (Figure 4H). Furthermore, antagomiR-107 induced Nrf2 cascade activation, causing Nrf2 protein stabilization (Figure 4I), HO1-NQO1 expression (Figure 4I–4J) and NOQ1 activity increase (Figure 4J). Together, these results show that miR-107 inhibition activated Nrf2 signaling and alleviated DEX-induced oxidative injury in osteoblasts.

### AMPK downstream Nrf2 cascade activation is required for antagomiR-107-induced osteoblast cytoprotection against DEX

**Figure 5:**
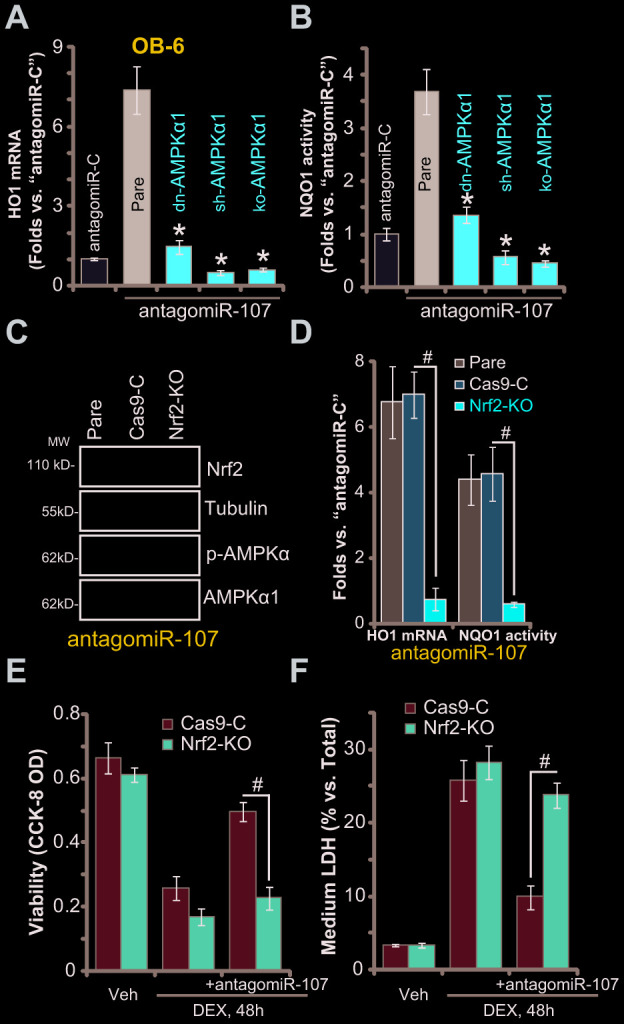
**AMPK downstream Nrf2 cascade activation is required for antagomiR-107-induced osteoblast cytoprotection against DEX.** Stable OB-6 cells, with the dominant negative AMPKα1 (dn-AMPKα1, T172A) construct, the lentiviral AMPKα1 shRNA (sh-AMPKα1), the CRISPR-Cas-9-AMPKα1 KO plasmid (ko-AMPKα1), as well as the parental control cells, were infected with antagomiR-107 lentivirus for 48h, relative *HO1 mRNA* expression (vs. “antagomiR-C” cells, **A**) and NQO1 activity (vs. “antagomiR-C” cells, **B**) were shown. Stable OB-6 cells, with the CRISPR-Cas-9-Nrf2 KO plasmid (“ko-Nrf2”) or CRISPR-Cas-9-control construct (“Cas9-C”), as well as the parental control cells were infected with antagomiR-107 lentivirus for 48h, expression of listed proteins was shown (**C**), relative *HO1 mRNA* expression and NQO1 activity (vs. “antagomiR-C” cells, **D**) were tested. Alternatively, cells were also treated with DEX (1 μM) or the vehicle control (“Veh”) for another 48h, cell viability (CCK-8 OD, **E**) and cell death (medium LDH release, **F**) were tested. Data were mean ± standard deviation (SD, n=5). * p<0.05 *vs.* “Pare” cells (**A**, **B**). ^#^ p<0.05 (**D**–**F**). Each experiment was repeated three times and similar results were obtained.

To test whether antagomiR-107-induced Nrf2 signaling activation is dependent on AMPK, the genetic strategies (see Figure 3) were applied again to block AMPK activation. As demonstrated, AMPK inactivation, by AMPKα1 dominant negative mutation, silencing or KO, almost blocked antagomiR-107-induced *HO1 mRNA* expression (Figure 5A) and NQO1 activity increase (Figure 5B) in OB-6 cells. These results imply that antagomiR-107-induced AMPK activation should be the upstream signaling for Nrf2 cascade activation. On the contrary, CRISPR/Cas9-induced Nrf2 KO (Figure 5C) did not affect antagomiR-107-induced AMPK activation (Figure 5C). Nrf2 KO, as expected, blocked antagomiR-107-induced *HO1 mRNA* expression and NQO1 activity increase (Figure 5D). Importantly, in Nrf2-KO OB-6 cells antagomiR-107 failed to efficiently inhibit DEX-induced viability reduction (Figure 5E) and cell death (Figure 5F), suggesting that Nrf2 cascade activation, downstream of AMPK, is required for antagomiR-107-induced osteoblast cytoprotection against DEX.

## DISCUSSION

The present study suggests that miR-107 is a novel and specific CAB39-tageting miRNA. RNA-Pull down assay results demonstrated that the biotinylated-miR-107 directly binds to *CAB39 mRNA* in OB-6 cells. Forced overexpression of miR-107, by infection of LV-pre-miR-107 or transfection of the wild-type miR-107 mimic, largely inhibited CAB39 3’-UTR luciferase activity and its expression in OB-6 cells and primary human osteoblasts. Contrarily, miR-107 inhibition, by antagomiR-107, increased CAB39 3’-UTR luciferase activity and its expression. Importantly, transfection of the mutant miR-107 mimics, with the mutations at the binding sites to CAB39 3’-UTR, failed to affect CAB39 3’-UTR luciferase activity and its expression in OB-6 cells. Therefore, miR-107 specifically targets and silences CAB39 in osteoblasts. miR-107 inhibition could be a novel strategy to boost CAB39 expression.

In osteoblastic cells or osteoblasts, forced activation of AMPK signaling has proven to be an efficient strategy to alleviate DEX-induced cytotoxicity [5, 20, 21, 30, 31]. AMPK activation could be achieved via pharmacological [5, 20] or genetic [21, 30, 31] methods in osteoblasts. Recent studies have implied that inhibition of CAB39-targeing miRNA can induce CAB39 upregulation to provoke downstream AMPK signaling activation, thus offering significant cyto-protection [26]. For example, Yang *et al.,* have shown that inhibition of CAB39-targeing miR-451, by antogomiR-451, activated AMPK signaling to inhibit oxygen glucose deprivation (OGD)-induced human umbilical vein endothelial cell (HUVEC) death [26]. Similarly, antogomiR-451 protected human gastric epithelial cells from ethanol via activation of AMPK signaling [25].

Here we discovered that inhibition of CAB39-targeting miR-107, by antagomiR-107, induced significant AMPK cascade activation, causing AMPKα1-ACC phosphorylation and AMPK activity increase. Functional studies demonstrated that antagomiR-107 potently attenuated DEX-induced cell death and apoptosis in OB-6 cells and human osteoblasts. Such osteoblast cytoprotective actions by antagomiR-107 were however abolished with AMPK in-activation, through genetic strategies including AMPKα1 dominant negative mutation, silencing or KO. We concluded that miR-107 inhibition by antagomiR-107 activated AMPK signaling to protect osteoblasts from DEX-induced cytotoxicity.

Our group and others have demonstrated that DEX treatment to the cultured osteoblastic cells/osteoblasts will provoke ROS production and significant oxidative stress, which are key mediators for cell death and apoptosis [31, 35, 36]. Contrarily, inhibition of oxidative injury can efficiently protect osteoblastic cells/osteoblasts from DEX-induced cytotoxicity [31, 35, 36]. Literatures have shown that forced activation of Nrf2 signalling, genetically or pharmacologically, can protect osteoblastic cells/osteoblasts from DEX-induced cytotoxicity [20, 30, 33–35, 37]. It has been shown that forced AMPK activation could stimulate Nrf2 cascade activation via different mechanisms [16, 17, 38–40].

In the present study we demonstrated that in OB-6 cells and primary human osteoblasts miR-107 inhibition induced Nrf2 signaling activation, causing Nrf2 protein stabilization, HO1-NQO1 expression and NQO1 activity increase. Importantly, blockage of AMPK signaling, by AMPKα1 mutation, silencing or KO, abolished antagomiR-107-induced Nrf2 activation in osteoblasts, suggesting that activation of AMPK is required for miR-107 inhibition-induced Nrf2 cascade activation. Further studies demonstrated that Nrf2 KO almost reversed antagomiR-107-induced osteoblast cytoprotection against DEX. Therefore, miR-107 inhibition provoked AMPK-dependent Nrf2 signaling, protecting osteoblasts from DEX-induced oxidative injury and cytotoxicity.

## MATERIALS AND METHODS

### Chemicals and reagents

DEX, polybrene, neomycin and puromycin were provided by Sigma Aldrich Chemicals (St Louis, Mo). From Gibco Co. (Shanghai, China) fetal bovine serum (FBS) and other cell culture reagents were obtained. Antibodies were all provided by Cell Signaling Technology (Danvers, MA). TRIzol and all RNA-associated reagents were purchased from Thermo-Fisher Invitrogen (Suzhou, China). All viral constructs, microRNA mimic (wild-type and mutants), and other sequences were provided by Shanghai Genechem Co. (Shanghai, China), unless otherwise mentioned.

### Cell culture

OB-6 human osteoblastic cells [6] and primary human osteoblasts [41, 42] were differentiated and cultured as described previously. The protocols of the study were approved by IACUC and Ethics committee of Nanjing Medical University.

### Forced expression or inhibition of miR-107

The lentiviral GV-369 vectors, encoding the pre-miR-107 sequence or the pre-miR-107 anti-sense sequence, were designed, synthesized and sequence-verified by Shanghai Genepharm Co. The construct and the lentivirus-packing plasmids (psPAX2 and pMD2.G) were co-transfected to HEK-293T cells, establishing pre-miR-107 expression lentivirus (LV-pre-miR-107) or the pre-miR-107 anti-sense lentivirus (antagomiR-107). The viruses were enriched, filtered, and added to cultured OB-6 cells or primary human osteoblasts (cultured in complete medium with polybrene) for 48h. When necessary puromycin was added in the complete medium for 6 days to select stable cells.

### qPCR

Total cellular RNA was extracted by TRIzol reagents. Detailed protocols of quantitative Real-time PCR (“qPCR”) were described previously [21, 43]. Quantization of targeted mRNAs was through the 2^ΔΔCt^ method (relative to GAPDH). miR-107 expression was analyzed by the TaqMan microRNA assay (Applied Biosystems, Shanghai, China), from 10ng of total RNA of each sample. Primers for the Nrf2 pathway genes and *GAPDH* were provided by Dr. Jiang at Nanjing Medical University [44, 45]. All other primers were listed in Table 1.

**Table 1:** Primers of the qPCR assay.

miR-107-F	5′-CAGCATTGTACAGGGCT-3′
miR-107-F	5′-GAACATGTCTGCGTATCTC-3′
U6 RNA-F	5′-CTCGCTTCGGCAGCACATATACT-3′
U6 RNA-R	5′-ACGCTTCACGAATTTGCGTGTC-3′
CAB39-F	5′-GAGCATGGCTGTTCTGGAAAAGC-3′
CAB39-R	5′- GCTACTGCTTCTGTCTGAGGCT-3′

### CAB39 3'-UTR luciferase activity assay

Human CAB39 3’-UTR, containing the putative binding sites of miR-107 (at position 1322-1229), was amplified, and inserted into the firefly luciferase reporter vector, pGL4.13 (luc2/SV40) (Promega) at the XbaI site and downstream from the stop codon of the luciferase gene. The plasmid, along with the Renillaluciferase reporter vector and pRL-SV40 (Promega), were co-transfected to OB-6 cells by Lipofectamine 2000. Afterwards, OB-6 cells were subjected to the applied genetic modifications, with CAB39 3'-UTR luciferase activity tested through a Promega kit [46].

### Cell functional assays

After the applied DEX treatment, cell viability assaying through Cell Counting Kit-8 (CCK-8, Dojindo Laboratories, Kumamoto, Japan) assay kit, cell death detection via the medium lactate dehydrogenase (LDH) release procedure, and cell apoptosis studies by TUNEL staining and caspase-3 activity assays were described in detail in our previous studies [3–5].

### miR mimic transfection

OB-6 osteoblastic cells were seeded into the six-well plates, transfected with 500 nM of the applied miR-107 mimic (wild-type and mutants) through Lipofectamine 2000 for 48h.

### Western blotting

Detailed protocols of Western blotting were described early [2–5]. For Western blotting assays, the same set of lysate samples were run in sister gels to test different proteins. The exact same amount of protein lysates, 40 μg lysates per lane, were loaded in each lane. The listed proteins were quantified and normalized to the loading control.

### AMPKα1 shRNA

The AMPKα1 shRNA lentiviral particles, reported early [21], were added to cultured osteoblasts. Stable cells were achieved by puromycin (1 μg/mL) selection. AMPKα1 knockdown (over 95% knockdown efficiency) was verified by Western blotting.

### AMPKα1 mutation

The dominant negative AMPKα1 (dn-AMPK-α1, T172A) construct, reported early [5, 21], was transfected to OB-6 cells [47]. Neomycin (1 μg/mL) was added to select table cells, with expression of dn-AMPK-α1 confirmed by Western blotting.

### AMPKα1 KO

The CRISPR/Cas9 AMPKα1-KO construct (from Dr. Pan at Shanghai Jiao Tong University [32]) was transfected to OB-6 cells via Lipofectamine 2000, with stable cells selected by puromycin. AMPKα1 KO in stable cells was confirmed by Western blotting.

### AMPK activity assay

From each treatment 150 μg of total cellular lysates were incubated with anti-AMPKα1 antibody (Santa Cruz Biotech, Shanghai, China). The AMPK activity was tested in the kinase assay buffer by adding AMP-[γ-^32^P] ATP mixture and AMPK substrate SAMS (HMRSAMSGLHLVKRR) peptide. Phosphocellulose paper was added to stop the reactions. The AMPK radioactivity was examined by a scintillation counter.

### Superoxide detection

Using a previously-described protocol [35, 36] OB-6 cells or human osteoblasts were seeded into the 96-well tissue-culturing plates, treated with DEX, and tested by a superoxide colorimetric assay kit (BioVision, Shanghai, China), with the superoxide’s absorbance tested at the 450 nm [44].

### Lipid peroxidation

Using a previously-described protocol [44] OB-6 cells or human osteoblasts were seeded into six-well plates, treated with DEX, and assayed by an lipid peroxidation kit (Abcam, Shanghai, China) through the thiobarbituric acid reactive (TBAR) method [44, 50].

### NQO1 activity

The assay of NQO1 activity, using menadione as the substrate, was performed via a previously-described protocol [51]. NQO1 activity in stimulated osteoblasts was normalized to that of untreated control cells.

### Nrf2 knockout

From Dr. Xu at Central South University [52] the lenti-CRISPR-GFP-Nrf2 knockout (KO) construct was obtained and transfected to OB-6 cells by Lipofectamine 2000. GFP-positive OB-6 cells were sorted by FACS, and monoclonal single stable cells achieved. Nrf2 KO was screened by qPCR and Western blotting assays.

### Mitochondrial depolarization

JC-1, a fluorescence dye, will aggregate in the mitochondria in stressed cells with mitochondrial depolarization, forming green monomers [53]. OB-6 cells or human osteoblasts were initially seeded into the 24-well plates, treated with DEX, and stained with JC-1 (5.0 μg/mL, Sigma). Cells were then tested via a fluorescence spectrofluorometer (Hitachi, Japan) at wavelength of 545 nm (green).

### Statistical analysis

The investigators were blinded to the group allocation. Experiments were repeated three times. Data were expressed as mean ± standard deviation (SD). Statistics were analyzed by one-way ANOVA through the Scheffe’s f-test. When compare significance between two groups, the two-tailed unpaired T test (Excel 2007) was applied. p values < 0.05 were considered statistically significant.
